# Allele-Specific Dual PCRs to Identify Members of the 27a Cluster of PPV

**DOI:** 10.3390/v14071500

**Published:** 2022-07-08

**Authors:** Vivien Tamás, István Mészáros, Ferenc Olasz, István Kiss, Zalán G. Homonnay, Preben Mortensen, Zoltán Zádori

**Affiliations:** 1 Veterinary Medical Research Institute, 1143 Budapest, Hungary; meszaros.istvan@vmri.hu (I.M.); olasz.ferenc@vmri.hu (F.O.); zadori.zoltan@vmri.hu (Z.Z.); 2 Scientific Support and Investigation Unit, Ceva-Phylaxia Co. Ltd., 1107 Budapest, Hungary; istvan.kiss@ceva.com (I.K.); zalan.homonnay@ceva.com (Z.G.H.); 3 Ceva Animal Health Ltd., 33500 Libourne, France; preben.mortensen@ceva.com

**Keywords:** *Ungulate protoparvovirus 1*, PPV-27a, PCR detection, porcine viruses, swine production

## Abstract

Porcine Parvovirus (PPV) is one of the most important infectious agents causing severe reproductive failure in pigs. In the last two decades a particular, a novel genotype emerged in Europe and PPV-27a was named as the prototype of this genetic cluster. It was suggested that members of the PPV-27a cluster may adversely influence effective vaccination against PPV. For a reliable updated 27a definition, we aligned 93 databank-deposited partial or full nucleotide and protein sequences of the VP2 of different PPV isolates. We confirmed that the 27a cluster could indeed be distinguished from other members of the species, however, some divergences were identified compared to earlier defined genetic markers. Based on genetic differences, we developed a dual allele-specific polymerase chain reaction for the easy and quick discrimination of members of the 27a cluster from other PPV strains. The detection limit of dual PCR was found <1.66 × 10^4^ copies/reaction. To sensitize and make it more user friendly, the method was further developed for qPCR application with fluorescent probes. Regarding the detection limit of the two PCRs (<1.66 × 10^4^ copies/reaction of the dual PCR versus <2.40 × 10^2^ copy/reaction of the dual qPCR), approximately two log improvement was achieved in the sensitivity of the method.

## 1. Introduction

*Ungulate protoparvovirus 1* (PPV) is the causative agent of SMEDI (stillbirth, mummification, embryonic death, and infertility) syndrome in swine [1,2]. PPV belongs to the genus Parvovirus in the family Parvoviridae and contains a 5 kb DNA genome with negative polarity. The left part of the genome, the large ORF1, and the small ORF3 encode three non-structural proteins (NS1, NS2, and NS3), while the right half of the genome encodes structural proteins (VP1, VP2, and VP3) [3].

PPV infection usually does not induce clinical signs in non-pregnant adult pigs or piglets. Strain virulence is determined by the severity of the reproductive failure it causes [4]. In pregnant sows, the outcome of PPV infection depends on the stage of gestation and the virulence and amount of the virus [5]. Investigations of pregnant sows indicate that 10,000 times more virus is needed from the NADL-2 strain to establish infection in foetuses than that from the more virulent NADL-8 strain [6].

Until the 2000s, the genetic changes of the PPV genome were not studied systematically, because PPV was anticipated to be very stable immunologically. Commercial vaccines developed from “ancient” strains provided full protection against all PPV variants [7]. However, later studies in the last decades concentrating on the genetic diversity of VP proteins from domestic pigs [8] and wild boars [9] revealed a broader spectrum of genetic variability of PPV than earlier thought. Variants were divided at least to seven clusters. A new variant, called PPV-27a (member of the B cluster [9] in other classification cluster D [8]), has become widespread in the German swine population. Variant 27a was suggested to be strongly virulent, immunologically “special”, cannot be neutralized by either homologous or heterologous sera, and commercial vaccines may not be fully protective against this genotype [7,8,10]. However, further direct experimental investigations of such vaccines were not able to confirm this idea [11,12,13,14]. Recently, much less significant serological differences of 27a from other PPV strains were reported [15] than were demonstrated earlier [7]. These conflicting data continue to keep strain 27a at the forefront of PPV research and there are both scientific and commercial interest for the rapid identification of 27a relative PPV strains.

One of the earliest characterizations of cluster 27a was described by Zimmermann et al. (2006) [16] based on the VP1 sequence alignment of 15 PPV isolates. A three amino acid pattern have been recognized to define members of cluster 27a [16] (though their numbering was not correct). In a later publication comparing 50 sequences, a four amino acid pattern could be recognized, although their explicit positions were not appropriately discussed [9].

The correct identification and quantification of 27a viruses are fundamental steps in ascertaining the biological significance of these variants. One of the main objectives of the research presented here was to update the definition of cluster 27a using the currently available VP2 sequences in the databank. The other goal of this study was to develop a reliable allele-specific polymerase chain reaction (asPCR) [17] method for the specific detection of members of the PPV 27a genetic cluster. According to the users’ requirements, the method can be utilized as a single tube dual PCR or a qPCR application.

## 2. Materials and Methods

### 2.1. Sequence Alignments and Phylogenetic Tree

Publicly available complete PPV VP2 DNA sequences were downloaded from GeneBank [18] and aligned with Multalin [19,20]. Phylogenetic tree was constructed by the Clustal Omega program with the neighbor joining method [21].

### 2.2. PPV Copy Number Determination

Viral copy numbers of the samples were determined as described earlier [22]. The viral DNAs were purified with High Pure Viral Nucleic Acid KIT (Roche, Basel, Switzerland) according to the manufacturer’s protocol. The qPCR reaction solution (25 μL) contained 16 µL water, 2.5 µL 10× DreamTaq Buffer (Thermo Fisher Scientific, Waltham, MA, USA), 2 µL dNTP mix (2.5 mM each), 1 µL template DNA from the supernatants, 0.2 µL DreamTaq DNA Polymerase (Thermo Fisher Scientific, Waltham, MA, USA), 1.25 µL 20× EvaGreenTM Dye (Biotium, Fremont, CA, USA), 1 µL forward (PPVF: 5′-CTTTAGCCTTGGAGCCGTGGA-3′), and 1 µL reverse (PPVR: 5′-AACTACCCTTACCTCTTGCTCTT-3′) primer (both 6.5 µM concentration).

The thermal reaction started with a pre-denaturation step at 95 °C for 5 min followed by 35 cycles (denaturation at 95 °C for 30 s, annealing at 62 °C for 30 s and elongation at 72 °C for 35 s) and finished with a post-elongation step at 72 °C for 5 min. The specificity of qPCR was verified with melting curve analysis. Viral copy numbers were calculated using a standard curve, for which purified amplicon was used as a template in 10-fold dilution. The PCR product was isolated from agarose gel with NucleoSpin^®^ PCR clean-up Gel extraction kit (Macherey-Nagel, Düren, Germany), following the manufacturer’s protocol. The optical density of the DNAs was measured with Nanodrop 2000 (Thermo Fisher Scientific, Waltham, MA, USA), and the copy number of the isolated fragment was calculated from the measured concentration.

### 2.3. Gradient PCRs for Selecting 27a Allele-Specific Primer Pairs

PS1 (T261CF: 5′-GAAACATACAAAAGAATACATGTACTAAAC-3′; C682GR: 5′-CTGTTTGTATTGAGTCTGTTATTTGTTC-3′) and PS2 (C682GF: 5′-ACCAACATACACTGGACAATCAG-3′; G1240TR: 5′-TTTGTATTTTGTAGGTTTAGTGGTGA-3′) primers were synthesized by Integrated DNA Technologies (Coralville, IA, USA). All 27a-specific primers were PAGE purified to ensure primer homogeneity and the presence of the ultimate 3′ end nucleotide, which is paramount for asPCR. PCRs were carried out with DreamTaq enzyme (it does not have 3′-5′ proofreading exonuclease activity (cannot digest the ultimate 3′ nucleotide of primers), but it has 5′-3′ exonuclease activity (can digest fluorescent probes) in the standard DreamTaq buffer in 25 μL volume after applying different Mg^2+^ ion concentrations. Since the factory-made DreamTaq buffer contains 2 mM Mg^2+^, EDTA (Thermo Fisher Scientific, Waltham, Massachusetts, USA) was added in different concentration to decrease the effective Mg^2+^ concentration. The following cycle conditions were applied using 0.4 μM for each primer, 0.4 mM for each dNTP and 2.5 U for DreamTaq polymerase: 95 °C 5 min, 95 °C 30 s, 55–72 °C 30 s, and 72 °C 35 s in 35 cycles, with different Mg^2+^ ion concentrations (1.0, 1.2, 1.4, 1.6, 1.8, 2.0, 2.5, and 3 mM Mg^2+^) adding 1, 0.8, 06, 0.4, and 0.2 mM EDTA or 0.5 and 1 mM MgCl_2_ (Thermo Fisher Scientific, Waltham, MA, USA), respectively.

### 2.4. PPV-Specific PCR

PPV-specific primers PPVSP1F (5′-CTGCAAAAAGAGCAAGAGGTAAG-3′) and PPVSP1R1 (5′-CCGTAGTCTTTTGCGTGTTC-3′) were used in 0.1, 0.2, or 0.4 µM final concentration alone or together with 0.4 µM PS1 primer set under the same PCR conditions as described above including 1.4 mM Mg^2+^ concentration with the following thermal profile: 95 °C 5 min, 95 °C 30 s, 61 °C 30 s, and 72 °C 50 s in 35 cycles.

### 2.5. 27a Allele-Specific Dual qPCRs

Fluorescent probes (27a-specific FAM labelled (probe1 (/56-FAM/TCAGCAACC/ZEN/TCACCACCAACCA/3IABkFQ) and probe2 (/56-FAM/TGGTACAAG/ZEN/ACGATGCACACACACA/3IABkFQ) and PPV amplicon- specific HEX labelled (probe3 (/5HEX/AGAACACGA/ZEN/CGAAGCCTACGACAA/3IABkFQ) and probe4 (/5HEX/TAATCCATC/ZEN/AGACGCCGCAGCA/3IABkFQ) with ZEN-Iowa black double quenchers were produced by Integrated DNA Technologies (Coralville, Iowa, USA). Gradient dual qPCRs were executed using the following primer combinations and conditions with 27a and non-27a PPV DNAs: 2,5 μL DreamTaq buffer, 2 μL 2,5 uM dNTP, 1 μL primer mix: 10 μM 27a-specific primers (PS1), 5 μM PPV-specific primers (PPVSP1F + PPVSP1R1), 5 μM of each probes A (probes 1, 3) or B (probes 2, 4), 6 μL 2,5 mM EDTA, 0,2 μL DreamTaq Hot Start DNA polymerase (5U), 12,3 μL water, 1 μL template. The temperature profile was as follows: 95 °C 5 min, 95 °C 30 s, 55–70 °C 30 s 72 °C 35 s in 35 cycles.

### 2.6. Allele-Validating PCR

Partial VP2 gene amplification was carried out using primers F3570 (5′-CTACCACAGAAGGAGACCAA-3′) and R4498 (5′-ATTGAAGTATACAATGATAGTAGT-3′) according to Cadar et al. (2012) [9]. PCR was performed in 50 μL PCR reactions consisting of 5 μL Dream Taq Buffer, 1 μL of each primer (10 pmol), 1 U of Dream Taq DNA Polymerase, 1.5 μL of dNTP (2.5 mM each, Promega, Madison, WI, USA), 1 μL MgCl_2_ solution (25 mM, Promega, Madison, WI, USA), 5 μL of DNA template, and ddH_2_O up to 50 μL. PCR conditions were 94 °C for 5 min, followed by 35 cycles of 94 °C for 30 s, 58 °C for 30 s, 72 °C for 2 min, and a final elongation step at 72 °C for 7 min.

## 3. Results

### 3.1. Definition of 27a Cluster

**Figure 1:**
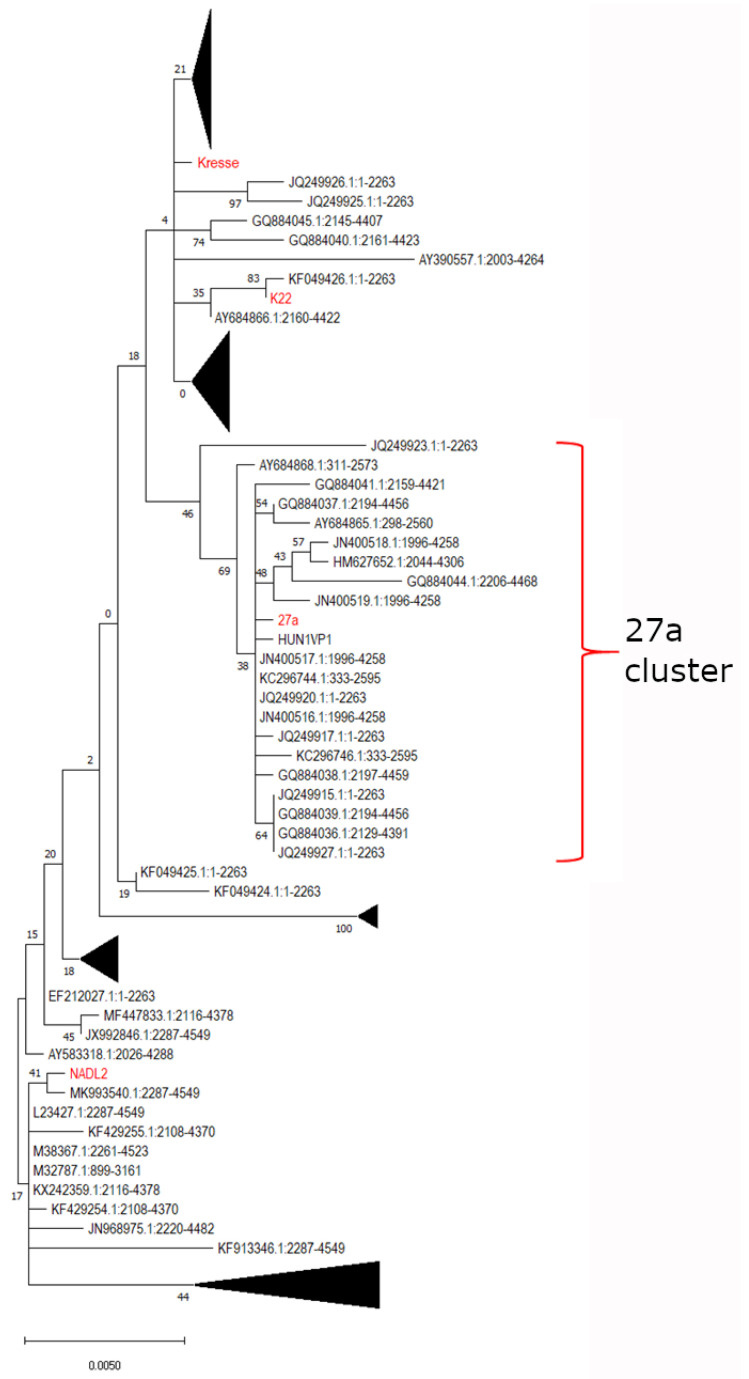
Phylogenetic tree of the full-length VP2 DNA sequence of 93 PPV isolates. The tree was constructed with the Mega X program (version 10.2.6.) using the maximum likelihood method with the Tamura–Nei model. Numbers indicate bootstrap values in percentage. Some branches of the tree are collapsed for a more visible representation. A few representative strains are highlighted in red.

For a reliable updated definition, we aligned 93 databank-deposited partial or full nucleotide and protein sequences of the VP2 of different PPV isolates and created a phylogenetic tree (Figure 1) based on the alignment of full-length nucleotide sequence of VP2 (Supplementary Figure S1). The 27a cluster could indeed be distinguished from other members of the species. However, two more genetic markers could be identified additionally to the three described earlier [16] (aa substitution 231 (obvious oversight, in reality 228), 419, and 436 (Supplementary Figure S2)).

**Figure 2:**
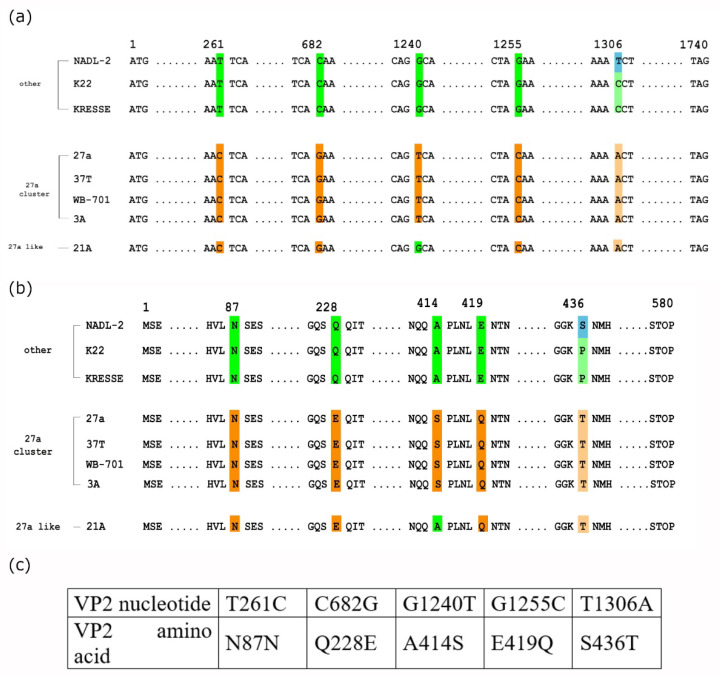
Mutations in the VP2 proteins defining the members of the 27a cluster. Nucleotide (**a**) and amino acid (**b**) differences between members of the 27a cluster and other representative members of the PPV species. Amino acids and nucleotides characteristic to members of 27a cluster are in dark orange; amino acids and nucleotides characteristic to 27a members but occasionally occurring in non-27a members are in light orange. Amino acids and nucleotides characteristic of non-27a members are in green and blue, respectively. PPV isolate (AY684868.1 strain 21A) containing only four 27a characteristic mutations, labelled as 27a-like virus. (**c**) Nucleotide and amino acid mutations characteristic to 27a viruses.

According to our investigations the 27a cluster is defined by the presence of five collectively occurring point mutations (T261C C682G G1240T G1255C T1306A) as a genetic marker in the coding gene of VP2 protein (Figure 2). Furthermore, the A447G silent mutation (V149V) (Supplementary Figures S1 and S2) is also present in the majority of the members of the 27a cluster. The occurrence of the five single nucleotide mutations seems to be linked; they can rarely be found alone or in combinations in lesser numbers (e.g., only two or three from these) (Supplementary Figure S1). Only one isolate with an incomplete “27a-like” pattern is reported (AY684868.1), containing only four of the linked five nucleotide mutations, and another one (AY684864.1) contained only three of the linked four amino acid mutations (Supplementary Figure S2). The significance of these exceptions remains to be investigated. The strong linkage of the four aa (228E, 414S, 419Q, and 436T) marker mutations may suggest a structural/functional cooperation among the coding nucleotides and/or their coded amino acids. All four amino acids are on the surface of the capsid [11,23]. Since it has been extensively documented that very few amino acid change on the capsid surface can alter the biological properties (e.g., host range, tissue specificity, and virulence) of parvoviruses [24,25,26,27], it might be assumed that these mutations have a role in the evolution of the supposedly more virulent nature of the cluster.

### 3.2. Development of a Dual Allele-Specific PCR

It is well known that templates with single nucleotide polymorphism can be best distinguished in an asPCR reaction by using PCR primers containing mismatches at their ultimate 3′ position [17]. The efficiency and distinguishing capability of such PCR depend on many factors, including the nature of the mismatch (the substituted and substituting nucleotides), the kinetics of association and dissociation of the different reaction components, and the effect of a mismatch on the stability of the duplex DNA formed in primer–template interactions (temperature, time of annealing and extension, ramp, size and Tm of the primers, Mg^2+^ ion concentration, and choice of polymerase) [28].

**Figure 3:**
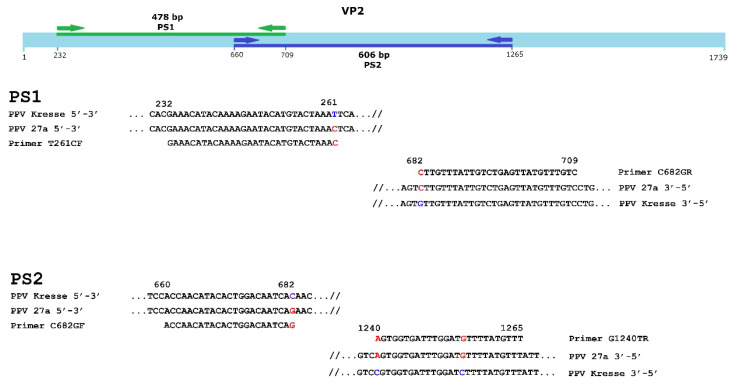
Rationale of the selection of 27a-specific asPCR primer pairs. PS1 (amplicon 478 bp) and PS2 (amplicon 606 bp) primer pairs are shown with the corresponding 27a and Kresse (as non-27a) sequences; 27a-specific nucleotides are in red.

In the case of the 27a cluster, the distance and nature of the distinguishing mutations and the surrounding sequences determine and restrict the selection of the suitable primer pairs for the development of a 27a-specific PCR. Considering the above-mentioned factors influencing the effectiveness of an asPCR reaction, two primer pairs, PS1 and PS2 (based on the ultimate 3′ T261C, C682G and C682G, and G1240T mutations) were selected to demonstrate the feasibility of a 27a-specific PCR (Figure 3).

**Figure 4:**
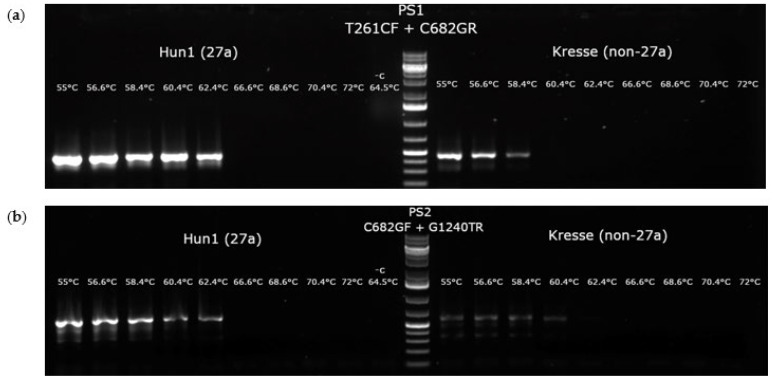
Gradient PCRs executed with PS1 and PS2 27a-specific asPCR primer pairs. PCRs were executed using Hun1 (member of 27a cluster) and Kresse (non-27a virus) templates with different Mg^2+^ ion concentration. Only the best performances are shown; (**a**) the result of a gradient PCR with PS1 primer pair at 1.4 Mg^2+^ (0.6 mM EDTA) concentration; (**b**) the result of a gradient PCR with PS2 primer pair at 1.8 Mg^2+^ (0.2 mM EDTA) concentration.

To compare the performance of the two primer pairs’, gradient PCRs (55–72 °C) were carried out with Hun1 (27a positive) and Kresse (27a negative) templates at different Mg^2+^ (1–3 mM) concentrations. PS1 primer pair was found to be superior to PS2 (Figure 3) in both yield and specificity for 27a, and it was selected for further work at 1.4 mM Mg^2+^ ion (0.6 mM EDTA) and at 61 °C annealing temperature concentration (Figure 4).

**Figure 5:**
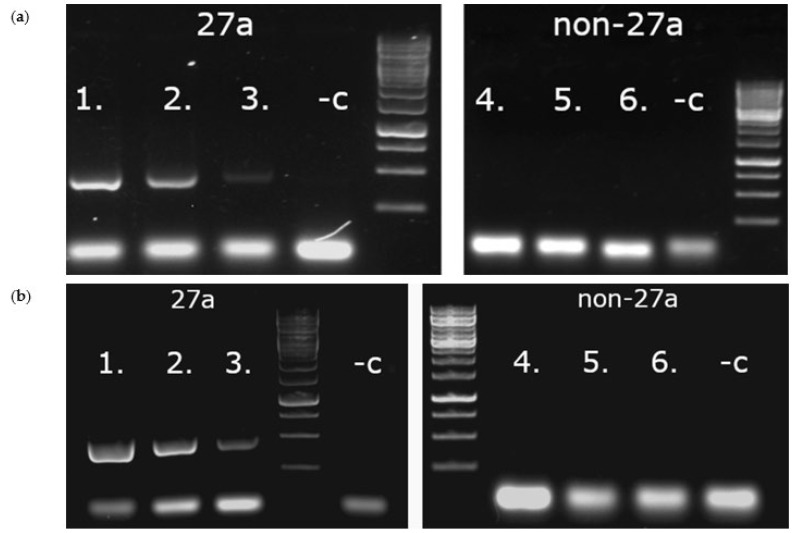
Efficiency of DreamTaq (**a**) and DreamTaq Hot Start (**b**) enzymes on different 27a and non-27a templates. Numbers 1–3 are three different 27a samples; 4–6 are three different non-27a samples; -c is negative control; markers are GeneRuler 1 kb DNA ladders.

The effect of hot start DreamTaq on selectivity and production was also investigated at the selected condition with a set of known 27a and non-27a samples. asPCR indeed proved to be 27a-selective and no visible difference was detected between the performances of the two enzyme mixes in their own buffers (Figure 5).

**Figure 6:**
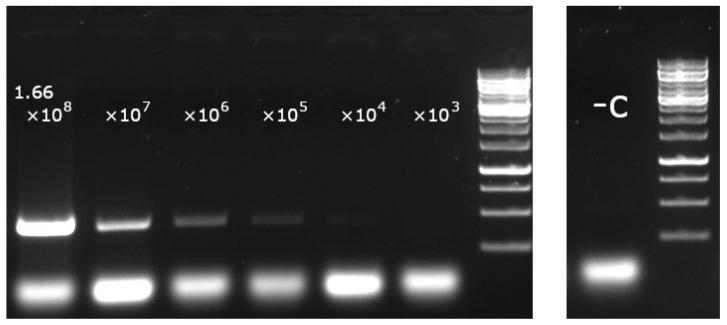
Detection limit of 27a-specific asPCR; 10-fold serially diluted 27a template was amplified by asPCR, and the products were loaded and run on an agarose gel. No visible band can be detected lower than 1.66 × 10^4^ copy/reaction at this dilution step.

To establish the detection limit of the asPCR, 1.66 × 10^8^ copy/ul 27a viral DNA template was 10-fold serially diluted and applied in different concentration at the selected condition. The detection limit for the 27a-specific PCR was found to be <1.66 × 10^4^ copies of the 27a DNA/reaction (Figure 6).

As the final goal of the project was to develop a single tube dual PCR (27a- and PPV-specific), we also tested a PPV-specific primer set (Psps) (PPVSP1F and PPVSP1R1) under the selected conditions. The advantage of such system is that it supplies an internal control for the presence of PPV DNA in the samples. If neither the PPV-specific nor the 27a-specific band can be detected, then there is no PPV DNA in the sample, or it is below the detection limit, or there is a problem with the PCR reaction.

**Figure 7:**
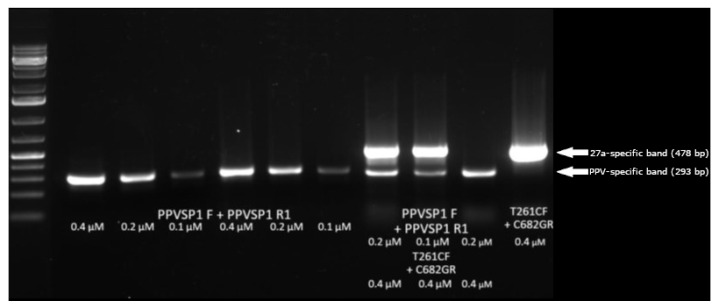
Combination of PPV and 27a-specific primer sets in a dual PCR. Lane 1, GeneRuler 1 kb+ ladder; Lane 2–7, Psps (PPVSP1F + PPVSP1R1 primers) alone in different concentrations with 27a and K22 (non-27a) templates; Lane 8–10, Dual PCR combining Psps and PS1 primer sets with 27a and K22 (non-27a) templates. Lane 11, PS1 primer set with 27a template. Primer concentrations are indicated on the lanes.

**Figure 8:**
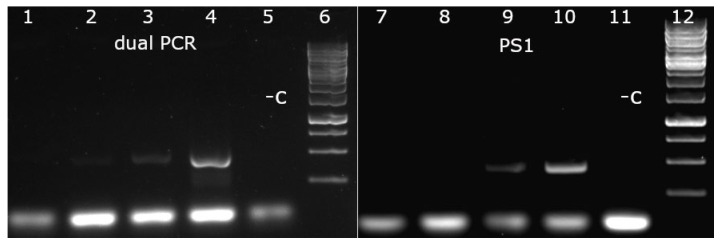
Comparison of 27a sensitivity of dual and 27a-specific PCRs. Lane 1 and 7, 0.55 × 10^4^; lane 2 and 8, 1.66 × 10^4^; lane 3 and 9, 4 × 10^4^; lane 4 and 10, 1.2 × 10^5^ copy/reaction 27a DNA respectively; lane 5 and 11, negative control; lane 6 and 12 Gene ruler 1 kb.

To establish the conditions for the single tube dual PCR, the efficiency of Psps alone and in combination with the PS1 27a-specific primer set (0.4 μM) was tested in different dilutions (0.1, 0.2, and 0.4 μM). The decrease in the concentration of Psps significantly influenced PCR efficiency when it was used alone to detect PPV; however, PPV-specific bands could still be detected when Psps (0.1, 0.2 μM) was used in combination with PS1. When 27a DNA was applied in high concentration (1.66 × 10^8^ copy/reaction), the intensity of 27a-specific band decreased somewhat in those reactions (Lane 8–9 of Figure 7) in which PS1 was used in combination with Psps compared to when used alone (Lane 11 of Figure 7). It could be the consequence of either the concentration decrease in GelRed stain by binding to the lower PPV-specific DNA band in the gel or of competition for the PCR reaction components in the test tube. At lower concentration DNA, the limit of 27a detection in the dual PCR remained the same as in the 27a-specific PCR < 1.66 × 10^4^ copy/reaction (Figure 8). Considering the best 27a sensitivity and acceptable PPV sensitivity, 0.4 μM 27a-specific primers and 0.2 μM PPV-specific primers were selected for further works. Dual PCR is optimized for 27a DNA detection, and PPV-specific primers underperform in the intentionally applied low concentration in order to minimally interfere with the 27a-specific reaction. In practice, PPV is usually determined from the infected foetus or other clinical samples with high sensitivity PPV-specific diagnostic PCR, so the decreased PPV-specific sensitivity of the dual PCR usually does not cause an unsolvable problem. The main function of the PPV-specific primers in the dual PCR is to supply an internal control for the reaction conditions and to confirm the presence of non-27a PPV DNA. Both PPV and 27a sensitivity of the system highly depends on the quantity and ratio of the two primer sets in the reaction; therefore, the preparation of a “primer master mix” is highly recommended. In particular, great care must be taken when new batches of primers are used, since the real quantity or concentration of the primers may vary significantly from the factory supplied data. In such cases, the recalibration of the primer sets in the master mix is crucial to ensure optimal performance and maximal reproducibility.

### 3.3. Development of qPCR Application

To sensitize and make it more user friendly, the method was further developed for qPCR application. Two internal probes were designed for each of the 27a (probe 1–2) and the PPV-specific amplicons (probe 3–4) with ZEN-Iowa black double quenchers and 5′ FAM and 5′ HEX fluorescent chromophores, respectively. For probe and temperature profile selection, two kinds of gradient dual qPCRs (A, probe 1 and 3; B, probe 2 and 4) were performed with 27a and NADL2 (non 27a) DNAs between 55 and 70 °C annealing temperatures.

Combination A was selected for further work due to its better performance with 27a DNA at near optimal (56–60.9 °C) annealing temperatures (Table 1). Considering potential temperature deviations among PCR machines, 58 °C was chosen for further work as the median of the optimal temperature range. The performance of the selected condition was further investigated with a 27a and a K22 (non-27a) DNA sample of known concentration. Since dual qPCR was optimized for 27a DNA detection and PPV-specific primers were intentionally applied in a lower concentration so as not to interfere with the 27a-specific reaction, PPV-specific PCR underperforms in 27a-positive samples. The sensitivity of the dual qPCR for 27a DNA proved to be <240 copy/reaction while for PPV DNA, in the case of 27a and non-27a templates, it was <2400 and <1700 copy/reaction, respectively (Table 2). Comparing the detection limit of the 27a dual PCRs (<1.66 × 10^4^ copy/reaction in the dual PCR versus < 240 copy/reaction of the dual qPCR), around a 2 order of magnitude improvement was identified.

### 3.4. Investigation of Clinical Samples with 27a-Specific PCRs

From clinical cases of reproductive failure characterised by any kind of foetal losses, including mummified or aborted foetuses, relevant tissue samples were collected. Western and Central European samples found to be PPV positive by diagnostic PCR were further investigated for the presence of 27a strain (Table 3). Both the dual PCR and dual qPCR were applied to distinguish 27a and non-27a PPV viruses in these field samples. Twenty-six of the total thirty-one samples investigated were found to be 27a positive with at least one of the methods. Samples originated from the same cases proved to be uniform regarding their genotype (27a or non-27a), indirectly confirming the reliability of the dual PCRs. To further investigate the consistency of the methods, a PCR was carried out on 20 samples and the resultant amplicons (921 bp amplicon) were sequenced to reveal the presence of three of the characteristic 27a mutations (G1240T, G1255C, T1306A). All 15 samples proven earlier to be 27a by the dual PCR-contained 1240T, 1255C, and 1306A patterns, while the five non-27a samples carried 1240G, 1255G, and 1306C nucleotides, confirming the high reliability of the method. Full genome sequences of three 27a isolates were also determined verifying again 27a genome characteristics. Interestingly, in eight of the fourteen cases, 27a type viruses were detected, suggesting a strong presence of these type of viruses in the investigated samples. However, we have to emphasize that our samples cannot be seen as representatives of the European pig or PPV population, and the premier goal of our investigation was to validate our PCR method on clinical samples.

## 4. Conclusions

There are conflicting data about the immunological features and the animal health importance of the 27a type viruses in the literature. Therefore, there is an ongoing scientific and commercial interest in the biology of these viruses. First, we redefined the distinguishing genetical features of the 27a cluster based on sequence alignments and phylogenetic analysis showing that five collectively occurring tightly linked point mutations (261C, 682G, 1240T, 1255C, and 1306A) distinguish the members of the cluster from other PPV variants. Exploiting two of the featuring point mutations (261C and 682G) allowed the utilization of an allele-specific primer set (PS1) for the development of a 27a-specific asPCR system. The 27a-specific sensitivity of the allele-specific primer pair was <1.66 × 10^4^ copies/reaction in both the single and dual PCR. The application of fluorescently labelled probes in a dual qPCR increased the sensitivity of the method to 27a DNA with around two magnitudes (<240 copy/reaction). The 27a-specific dual PCR and qPCR were validated on clinical samples by partial VP2 and full genome sequencing and found them to be 100% reliable.

The use of allele-specific PCR primers enables the quick differentiation of 27a-type viruses from other PPV genotypes. According to the users’ preference, any of the PCRs developed based on these can be used as diagnostic tools in veterinary practice. In addition, the application of this versatile PCR system can facilitate field studies that may contribute to the unambiguous assessment of the prevalence of the virus and to the better understanding of the biology of 27a.

## Figures and Tables

**Table 1:** Gradient dual qPCRs using FAM and HEX labelled probes for 27a and PPV-specific detection. In the table, the actual annealing temperatures of the gradient are shown with the corresponding Ct values. The quantity of the 27a and non-27a (NADL-2) DNA were arbitrary chosen, but the same amounts were used for each kind of reaction. FAM and HEX channels represent 27a and PPV-specific PCRs, respectively.

(a) A Set (Probe 1 + 3)						(b) B Set (Probe 2 + 4)					
T [°C]	Sample	Ct Average	Ct SD	Ct Average	Ct SD	T [°C]	Sample	Ct Average	Ct SD	Ct Average	Ct SD
56.0	27a	15.63	0.09	16.49	0.70	56.0	27a	17.05	0.07	16.16	0.08
58.0	27a	15.72	0.49	17.20	0.21	58.0	27a	17.12	0.07	16.41	0.10
60.9	27a	16.16	0.26	17.31	0.92	60.9	27a	17.25	0.08	16.72	0.11
64.5	27a	N/A	N/A	26.56	0.66	64.5	27a	20.73	0.19	18.06	1.65
67.5	27a	N/A	N/A	N/A	N/A	67.5	27a	N/A	N/A	N/A	N/A
69.2	27a	N/A	N/A	N/A	N/A	69.2	27a	N/A	N/A	N/A	N/A
56.0	NADL-2	N/A	N/A	17.29	0.81	56.0	NADL-2	N/A	N/A	17.21	0.11
58.0	NADL-2	N/A	N/A	17.52	0.32	58.0	NADL-2	N/A	N/A	17.33	0.13
60.9	NADL-2	N/A	N/A	17.93	1.06	60.9	NADL-2	N/A	N/A	17.56	0.12
64.5	NADL-2	N/A	N/A	25.45	2.56	64.5	NADL-2	N/A	N/A	19.13	0.14
67.5	NADL-2	N/A	N/A	N/A	N/A	67.5	NADL-2	N/A	N/A	N/A	N/A
69.2	NADL-2	N/A	N/A	N/A	N/A	69.2	NADL-2	N/A	N/A	N/A	N/A

**Table 2:** Sensitivity of the dual qPCR using 27a (**a**) and non-27a (K22) (**b**) DNA. One μL of 27a (2.390 × 10^9^) and non-27a DNA (1.705 × 10^8^) was used for the reactions in ten-fold dilutions.

(a)						(b)					
		FAM Ct		HEX Ct				FAM Ct		HEX Ct	
Sample	Dilution	Average	SD	Average	SD	Sample	Dilution	Average	SD	Average	SD
27a	1x	9.48	0.88	12.52	1.45	K22	1x	N/A	N/A	11.76	0.57
27a	10x	12.29	1.36	15.62	1.75	K22	10x	N/A	N/A	16.18	0.82
27a	10^2^x	16.53	1.17	19.91	2.27	K22	10^2^x	N/A	N/A	23.94	0.74
27a	10^3^x	20.44	1.56	23.97	2.04	K22	10^3^x	N/A	N/A	28.21	0.63
27a	10^4^x	23.52	2.38	27.75	1.64	K22	10^4^x	N/A	N/A	31.98	0.71
27a	10^5^x	26.88	2.30	30.68	2.55	K22	10^5^x	N/A	N/A	33.56	0.72
27a	10^6^x	30.81	2.12	32.00	2.64	K22	10^6^x	N/A	N/A	N/A	N/A
27a	10^7^x	32.66	2.14	N/A	N/A	K22	10^7^x	N/A	N/A	N/A	N/A
27a	10^8^x	N/A	N/A	N/A	N/A						

**Table 3:** Results of the different diagnostic methods on various samples collected from Western and Central Europe.

Case	Saw	Sample	Organ	PPV/27a PCR	PPV/27a Real-Time PCR	Partial VP2 Sequence Analysis	Full Genome Sequencing
a	I	1	heart of foetus	27a	not tested	27a	27a
	II	1	kidney of foetus	27a	27a	27a	27a
	III	1	heart of foetus	27a	not tested	27a	not tested
b	I	1	aborted foetus	27a	27a	27a	not tested
c	I	1	mummified foetus	27a	27a	27a	27a
	II	1	mummified foetus	27a	27a	not tested	not tested
		2	mummified foetus	27a	27a	not tested	not tested
	III	1	mummified foetus	27a	27a	not tested	not tested
		2	mummified foetus	27a	27a	not tested	not tested
	IV	1	mummified foetus	27a	27a	not tested	not tested
		2	mummified foetus	27a	27a	not tested	not tested
	V	1	mummified foetus	27a	27a	not tested	not tested
	VI	1	mummified foetus	27a	27a	not tested	not tested
		2	mummified foetus	27a	27a	not tested	not tested
d	I	1	foetus	27a	not tested	27a	not tested
	II	1	foetus	27a	not tested	27a	not tested
	III	1	foetus	27a	not tested	27a	not tested
e	I	1	lung of foetus	27a	not tested	27a	not tested
	II	1	lung of foetus	27a	not tested	27a	not tested
	III	1	lung of foetus	27a	not tested	27a	not tested
f	I	1	mummified foetus	27a	not tested	27a	not tested
g	I	1	mummified foetus	27a	27a	27a	not tested
	II	1	mummified foetus	27a	27a	27a	not tested
	III	1	mummified foetus	27a	27a	not tested	not tested
	IV	1	mummified foetus	27a	27a	not tested	not tested
h	I	1	foetus	27a	not tested	27a	not tested
i	I	1	foetus	not 27a	not tested	not 27a	not tested
j	I	1	foetus	not 27a	not tested	not 27a	not tested
k	I	1	foetus	not 27a	not tested	not 27a	not tested
l	I	1	foetus	not 27a	not tested	not 27a	not tested
m	I	1	foetus	not 27a	not tested	not 27a	not tested

## Data Availability

The data presented in this study are available in the manuscript. The accession numbers of the sequences from GenBank used for our investigations can be found in the Supplementary Figures.

## Supplementary Materials

The following supporting information can be downloaded at: https://www.mdpi.com/article/10.3390/v14071500/s1, Supplementary Figure S1: Nucleotides characteristic to members of 27a cluster; Supplementary Figure S2: Amino acids characteristic to members of the 27a cluster.
