# Supplementary material for: Allele-Specific Dual PCRs to Identify Members of the 27a Cluster of PPV
Source: Viruses. 2022 Jul 8;14(7):1500. doi: 10.3390/v14071500 (PMC9324464; doi:10.3390/v14071500)
Supplement: Supplementary file 1 [file viruses-14-01500-s001.zip › Figure S1.pdf]

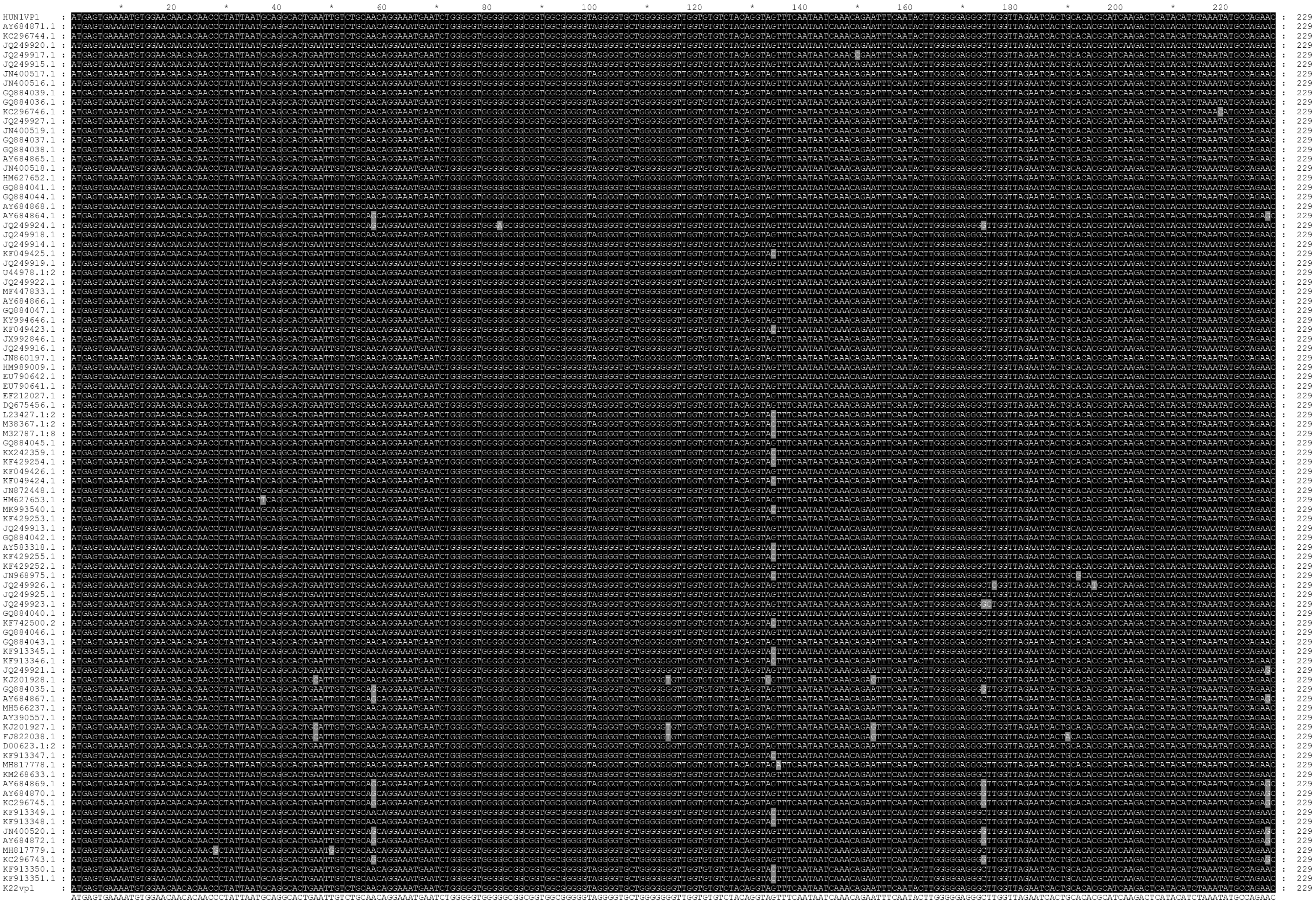

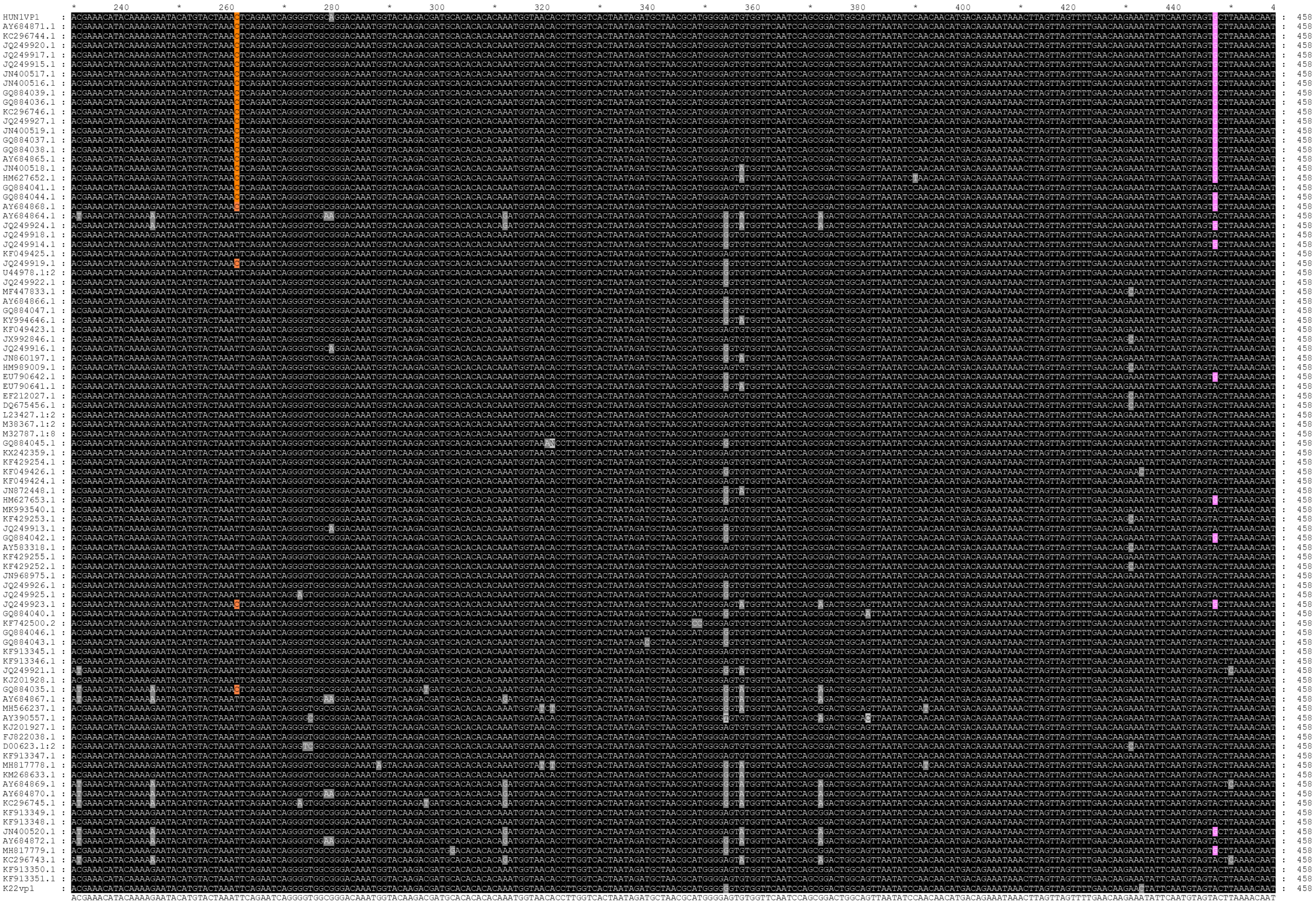

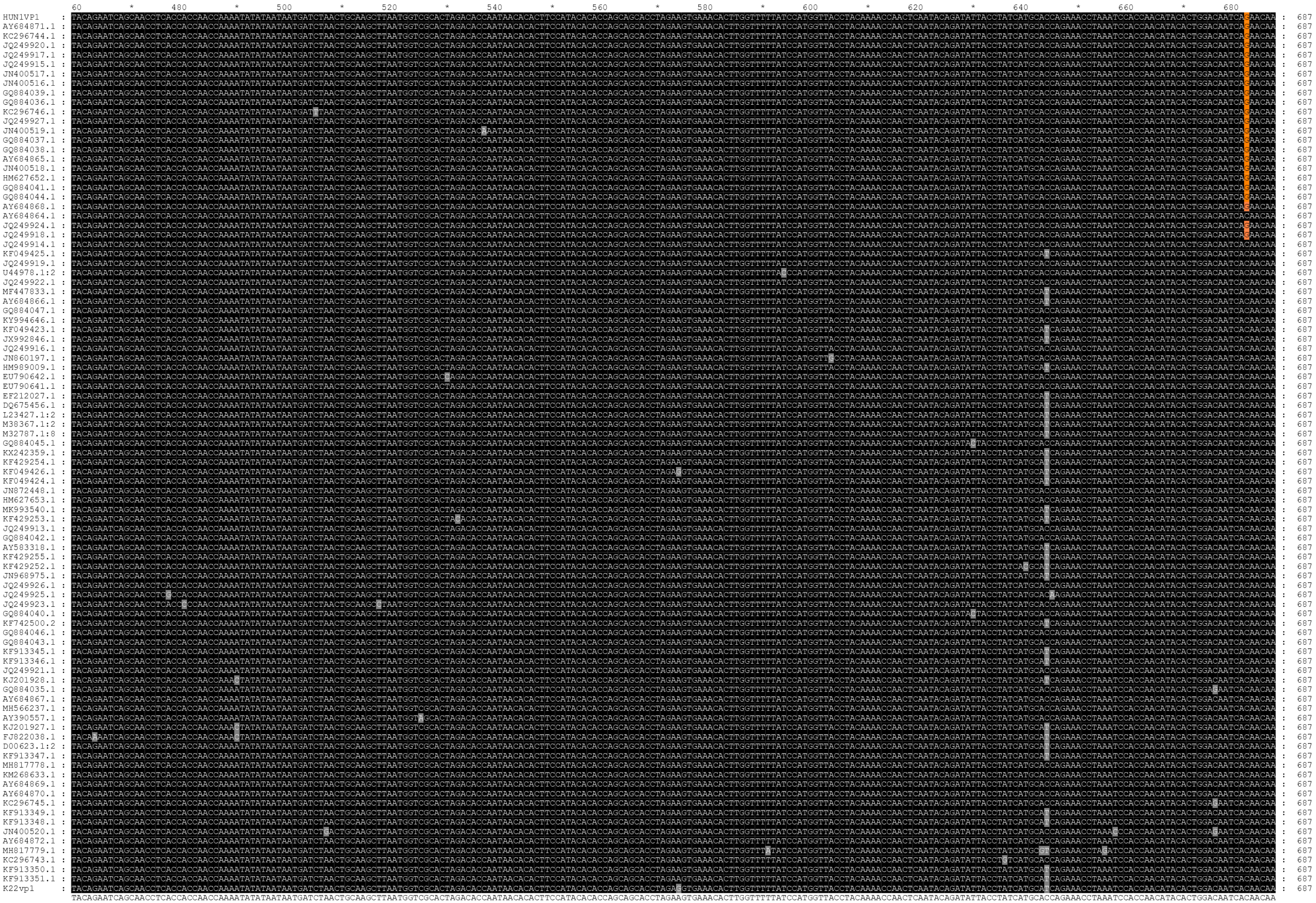

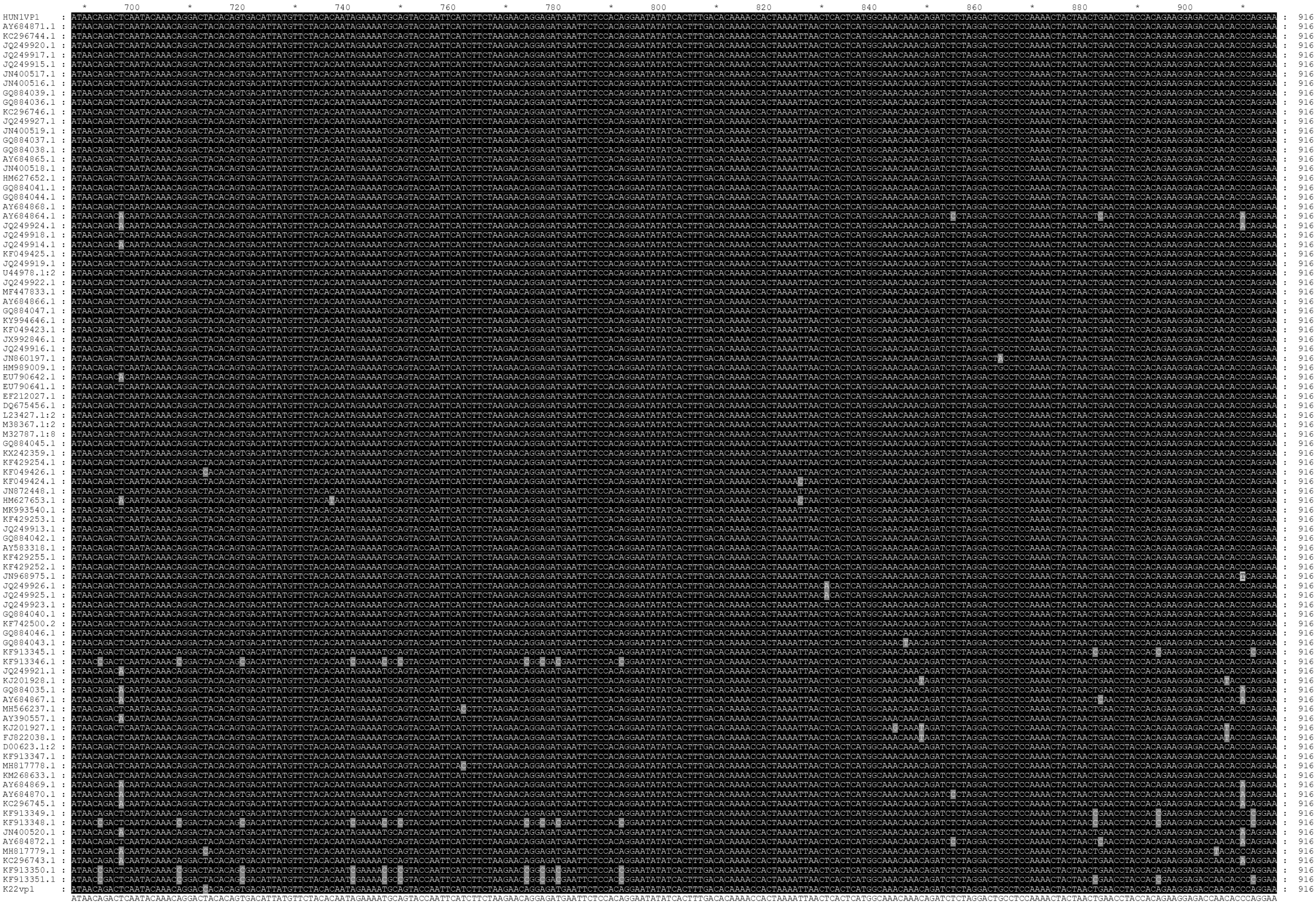

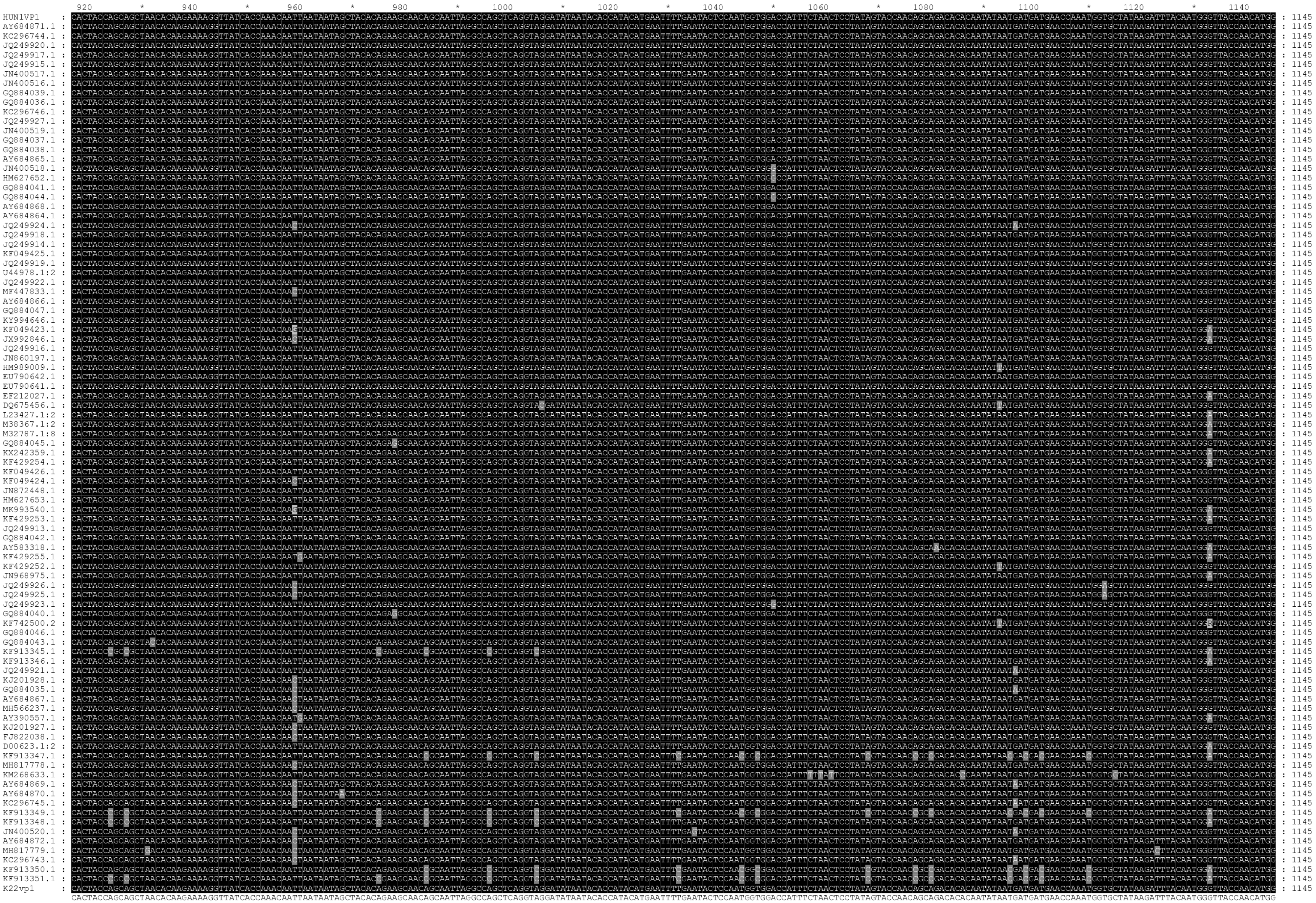

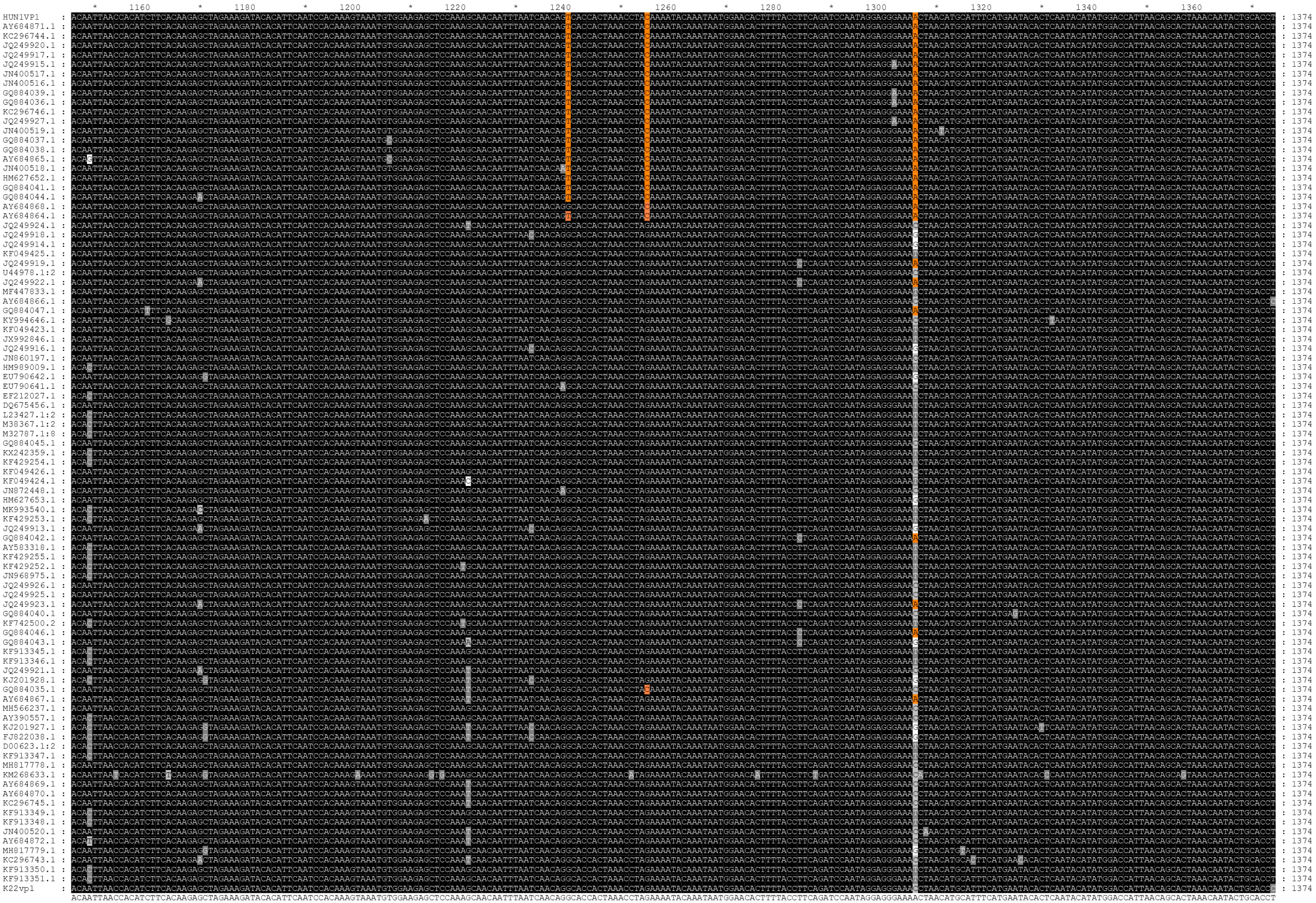

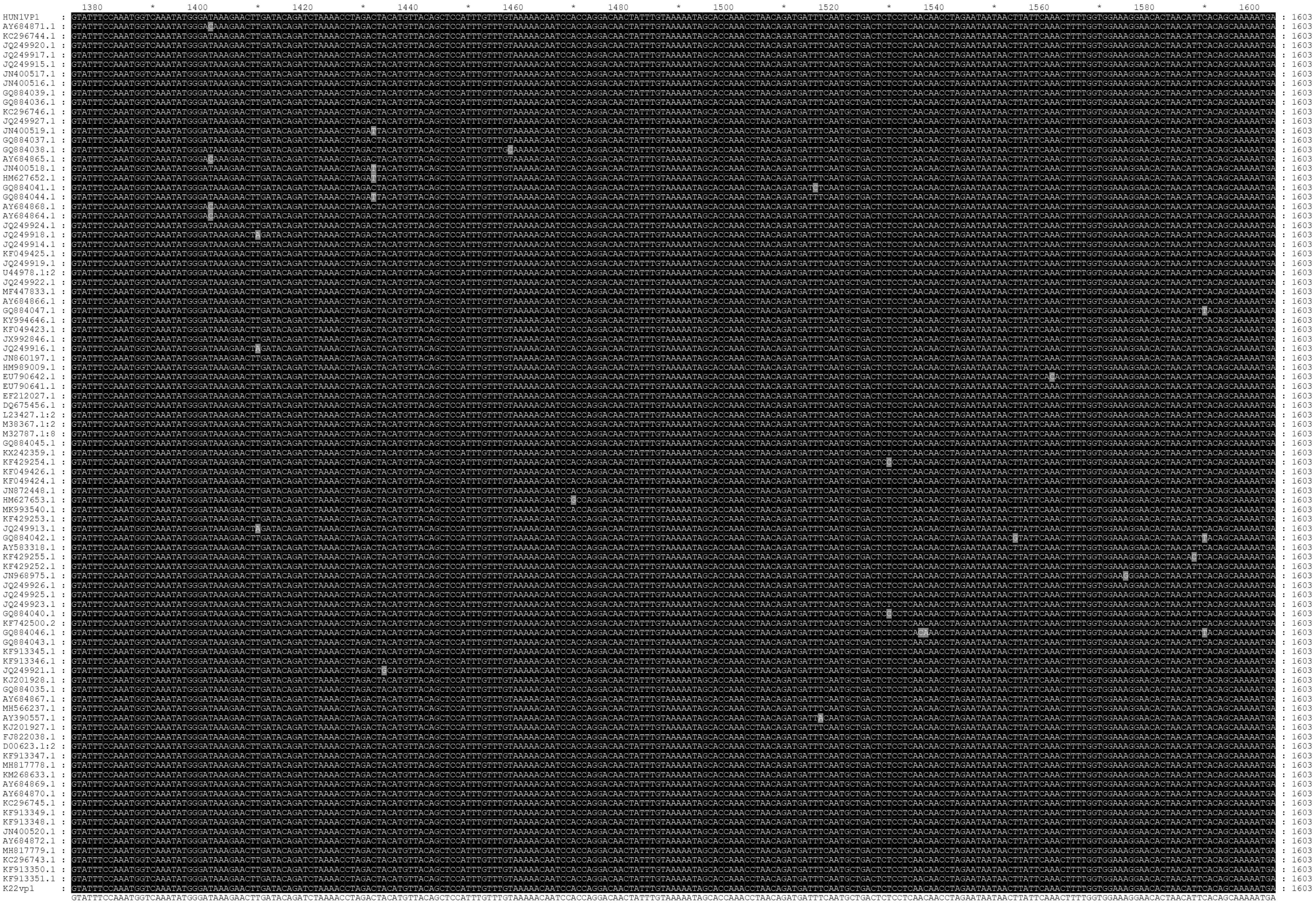

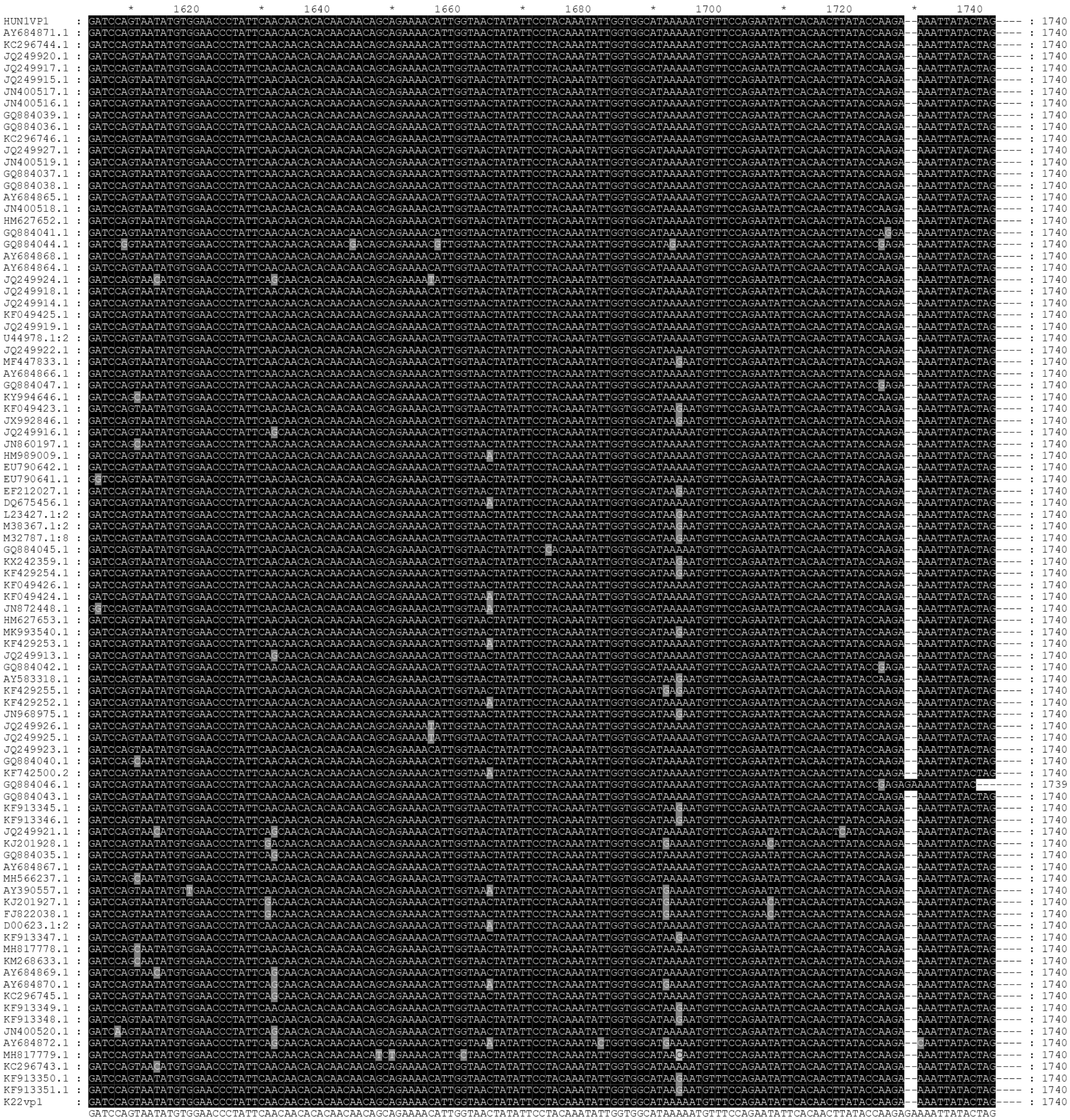

Supplementary figure 1.
Nucleotides characteristic to members of 27a cluster are in dark orange.
